# Alix and Syntenin-1 direct amyloid precursor protein trafficking into extracellular vesicles

**DOI:** 10.1186/s12860-020-00302-0

**Published:** 2020-07-30

**Authors:** Allaura S. Cone, Stephanie N. Hurwitz, Gloria S. Lee, Xuegang Yuan, Yi Zhou, Yan Li, David G. Meckes

**Affiliations:** 1 Department of Biomedical Sciences, Florida State University College of Medicine, 1115 West Call Street, Tallahassee, FL 32306-4300 USA; 2 Department of Chemical and Biomedical Engineering, Florida State University, Tallahassee, FL USA

**Keywords:** Extracellular vesicle, Exosomes, Alzheimer dementia, Neurodegeneration, Multivesicular bodies, Protein trafficking

## Abstract

**Background:**

Endosomal trafficking and amyloidogenic cleavage of amyloid precursor protein (APP) is believed to play a role in the neurodegeneration observed in Alzheimer’s disease (AD). Recent evidence has suggested that packaging and secretion of APP and its amyloidogenic cleaved products into small extracellular vesicles (EVs) may facilitate uptake of these neurotoxic factors during disease progression. However, the molecular mechanisms underlying trafficking of APP into EVs are poorly understood.

**Results:**

In this study, the mechanism and impact of APP trafficking into extracellular vesicles (EVs) were assessed by a series of inducible gene knockdowns. We demonstrate that vesicle-associated proteins Alix and Syntenin-1 are essential for proper subcellular localization and efficient EV secretion of APP via an *endosomal sorting complexes required for transport* (ESCRT)-independent pathway. The neurotoxic C-terminal fragment (CTFβ) of APP is similarly secreted in association with small vesicles. These mechanisms are conserved in terminally differentiated neuron-like cells. Furthermore, knockdown of Alix and Syntenin-1 alters the subcellular localization of APP, sequestering the precursor protein to endoplasmic reticulum and endolysosomal compartments, respectively. Finally, transfer of small EVs containing mutant APP confers an increase in reactive oxygen species production and neurotoxicity to human induced pluripotent stem cell-derived cortical neurons and naïve primary neurons, an effect that is ameliorated by Alix and Syntenin-1 depletion.

**Conclusions:**

Altogether these findings elucidate a novel mechanism for understanding the intracellular trafficking of APP and CTFβ into secreted extracellular vesicles, and the resultant potential impact on neurotoxicity in the context of Alzheimer’s disease amyloidopathy.

## Background

Alzheimer’s disease (AD) remains the major cause of dementia among the elderly, yet despite significant attention to the rising burden of disease, little is certain with regards to the mechanism of AD development and progression. Despite questions surrounding the mechanisms of neurodegeneration in AD, abundant research has pointed to the involvement of amyloid beta (Aβ) in producing neurotoxicity seen in the course of the developing dementia. Early in vitro work has demonstrated that Aβ fibrils, especially those formed by aggregated Aβ42 monomers, are highly toxic to neuronal cells in culture [1, 2]. A number of pathogenic mutations in amyloid precursor protein (APP) that lead to increased Aβ production have been identified, including the Swedish mutation (K670N/M671L) at the amino terminus of the Aβ region and the Indiana mutation (V717F), among others [3–5]. Subsequent in vivo transgenic mouse lines have been developed which overexpress amyloidogenic variants of human APP [6]. These mutations lead to increased amyloidogenic cleavage of APP, significant amyloid deposition and inflammatory response by 4 to 6 months, and detectable impairment on spatial memory testing. The Alzheimer’s-like phenotype of APP mutant mice has generated much interest in understanding the physiological role of APP, in addition to its processing and intracellular trafficking.

The *APP* gene is located on human chromosome 21q21.3 and gives rise to three major isoforms, with *APP*695 (a 695 amino acid protein) most predominately expressed in neurons [7, 8]. The full-length protein is a single pass transmembrane protein with an intracellular C-terminus, and is synthesized in the endoplasmic reticulum before transportation through the Golgi and trans-Golgi network into secretory vesicles to the plasma membrane. On the cell surface, APP can be proteolytically cleaved by α-secretase then γ-secretase to generate a soluble ectodomain product (sAPPα), which may be neuroprotective [9–11]. Unprocessed APP is internalized into the endosomal compartment, which contains β-secretase enzymes that cleave APP to produce sAPPβ and Β-secretase derived C-terminal fragment of APP (CTFβ). Next, γ-secretase cleaves CTFβ to produce CTFγ and Aβ [12, 13]. Recent research identifying new secretases such as δ-secretase, η-secretase, and meprin-β have added to the intricacy of APP processing [14]. These downstream derivatives have been a topic of recent research, particularly with regards to elucidating the role these different metabolites play in both AD and healthy cells. Interestingly, evidence suggests that trafficking of APP into lipid rafts and localization to acidic endosomes, where the β-secretase BACE is present, is necessary for amyloidogenic processing [15–19].

More recent studies have demonstrated that APP and its neurotoxic β-secretase derived catabolites, CTFβ and Aβ, are trafficked into secreted extracellular vesicles (EVs), including endosomal-derived exosomes [20–23]. It is worth noting that CTFβ has also been found to lead to inflammation and synaptic dysfunction [24] and may accumulate before Aβ peptides in the brains of AD mice and human patients [25, 26]. It has been suggested that CTFβ is particularly important in the preliminary stages of AD progression, and accumulation may lead to early lesions in the hippocampus [25]. With regards to EV secretion, intercellular transport of APP or the β-secretase derived catabolites likely has a harmful effect on naïve neurons [21], while inhibition of EV release may result in decreased amyloid plaque load present in AD mouse models [27]. Decreased release of small EVs has been found to augment oligomerized CTFβ accumulation in the endolysosomal compartments of cells [28]. This intracellular aggregation could lead to endosomal dysfunction, which is an early feature of AD [24].

Despite the significance of EV-associated secretion of APP, how APP is packaged into these vesicles is largely unknown. In general, several molecular pathways of small EV formation and cargo packaging are regarded, including processes involving *endosomal sorting complexes required for transport* (ESCRT) complexes [29–31]. The ESCRT pathway consists of four distinct protein complexes (ESCRT -0,-I,-II, and -III) in addition to several ESCRT-associated proteins (Alix, Vps4a, and Vta1) [32, 33]. Briefly, the ESCRT-0 complex is comprised of Hepatocyte growth factor-regulated tyrosine kinase substrate (Hrs) and Signal transducing adaptor molecule (Stam) proteins which bind and sequester ubiquitinated cargo for delivery to multivesicular bodies (MVBs) [31]. Hrs is responsible for recruitment of the ESCRT-I protein Tsg101, and the ESCRT-II complex subsequently assembles to guide MVB biogenesis and membrane budding, forming intraluminal vesicles later secreted as exosomes. ESCRT-associated protein Alix aids in drafting the ESCRT-III complex to the endosomal membrane to guide membrane scission and vesicle formation in MVBs [31]. Additional evidence suggests Alix also interacts with syndecans and an adaptor protein Syntenin-1, which facilitate vesicle protein trafficking through binding of syndecan, a type of heparan sulphate proteoglycan, with numerous ligands in an ESCRT-independent manner [34, 35]. In other scenarios, vesicle production and cargo packaging may instead be dependent on tetraspanin-mediated biogenesis or ceramide-driven membrane budding [36–40].

Here, we corroborate previous research [20–23] showing enrichment of wild-type and Swedish mutant amyloid precursor protein (APP^WT^ and APP^swe^) and its CTFβ metabolite into small EVs from HEK293 cells, in addition to differentiated SH-SY5Y neuronal cells. Through gene knockdown (KD) analyses, we further demonstrate that secretion of these AD-associated proteins is dependent upon an Alix- and Syntenin-1 mediated mechanism of vesicle cargo sorting. Cellular localization of APP is largely disrupted following Alix and Syntenin-1 KD, suggesting the importance of the previously recognized Alix-Syntenin-1 pathway in trafficking the amyloid precursor protein within cells. Finally, we reveal that Alix and Syntenin-1 depletion ameliorates the reactive oxygen species production and neurotoxicity observed following transfer of APP- and CTFβ- containing EVs onto naïve neuronal cells. Altogether these findings elucidate a novel mechanism for APP sorting, processing, and secretion from cells, which likely has downstream consequences in the context of AD progression.

## Results

### Mutant amyloid precursor protein mutant is secreted into small EVs

**Fig. 1:**
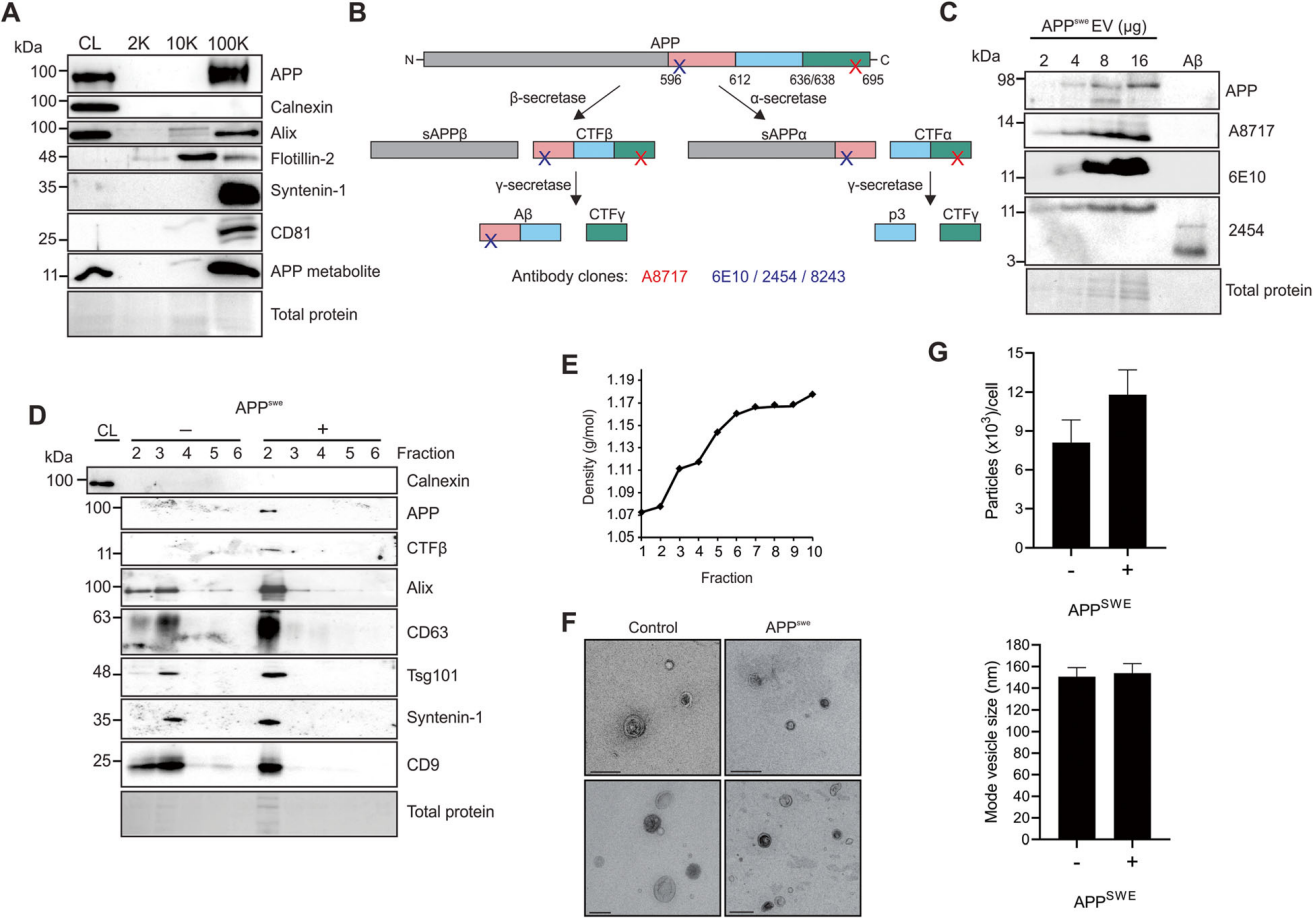
Amyloid precursor protein and amyloid beta are packaged into small extracellular vesicles. **a** Immunoblot analysis of HEK293 cell-derived EVs harvested by modified differential centrifugation following APP^swe^ transfection. **b** Schematic of APP proteolytic processing and epitope binding by several commercial antibody clones targeting APP metabolites. **c** EV protein was titrated and probed by several antibodies recognizing the C-terminus of APP (A8717) or N-terminus of Aβ/CTFβ (6E10, 2454) in comparison to purified oligomerized Aβ. **d** EVs were enriched by polyethylene glycol incubation and ultracentrifugation before subsequent purification and fractionation on an iodixanol density gradient. Equal volume was loaded for immunoblot analysis. One μg of cell lysate was run to demonstrate depletion of Calnexin in isolated EV fractions. Blots are representative images from at least three repeated independent experiments. **e** Densities of gradient separated fractions were estimated by measuring refractive indices of fractions with a refractometer. **f** Electron microscopy of gradient purified EVs (scale bar = 200 nm), and **g**) nanoparticle tracking analysis of small EVs (100 K g pellet) secreted from control and APP^swe^ transfected cells averaged across three biological replicates

Amyloid precursor protein harboring the Swedish mutation has previously been demonstrated to be secreted into EVs, and transmitted intercellularly [21]. Here, we demonstrate the co-enrichment of APP^swe^ and other small EV proteins in vesicles following ultracentrifugation at 100,000 g (Fig. 1a). Enriched EVs were devoid of Calnexin, an intracellular endoplasmic reticulum protein. Interestingly, APP and its β-secretase cleaved metabolite were not present in large vesicles pelleted at 2000 g, and only trace amounts of APP metabolites were isolated in medium-sized Flotillin-2 enriched vesicles pelleted at 10,000 g.

It is well documented that antibodies targeting the secretase-cleaved products may display cross-reaction, particularly between Aβ, CTFα, and CTFβ. To distinguish the identity of the predominant APP metabolite seen in EVs, several antibodies targeting either the C-terminus of APP, or the N-terminus of Aβ were used (Fig. 1b). Titrated EV protein showed increasing levels of a metabolite between 11 and 14 kilodaltons (kDa) that was detectable with all antibodies used (Fig. 1c). Comparison to purified Aβ oligomer detected between 4 and 5 kDa revealed CTFs to be the most prevalent metabolite in EVs rather than Aβ. Furthermore, primary probing with antibody clone 6E10 which binds the N-terminal of Aβ or CTFβ detected a band of similar molecular mass, indicating that the identify of this metabolite was most likely consistent with CTFβ rather than CTFα (Fig. 1b and c). It is possible that other cleaved products were present in EVs at lower levels. The remainder of immunodetection of CTFβ was performed using antibody clones 2454 and 8243.

Small EVs were further purified on an iodixanol density gradient, where APP was found to co-migrate with proteins associated with small EVs, including Tsg101, CD63, CD9, Syntenin-1, and Alix, to fractions previously demonstrated to be consistent with the density of exosomal-enriched isolates [40–43] (Fig. 1d-e). Vesicles enriched from cells expressing the mutant APP demonstrated no significant difference in morphology, size, or quantity compared to EVs from normal control cells by electron microscopy (Fig. 1f) and nanoparticle tracking (Fig. 1g).

### Alix and Syntenin-1 guide vesicle packaging of APP

**Fig. 2:**
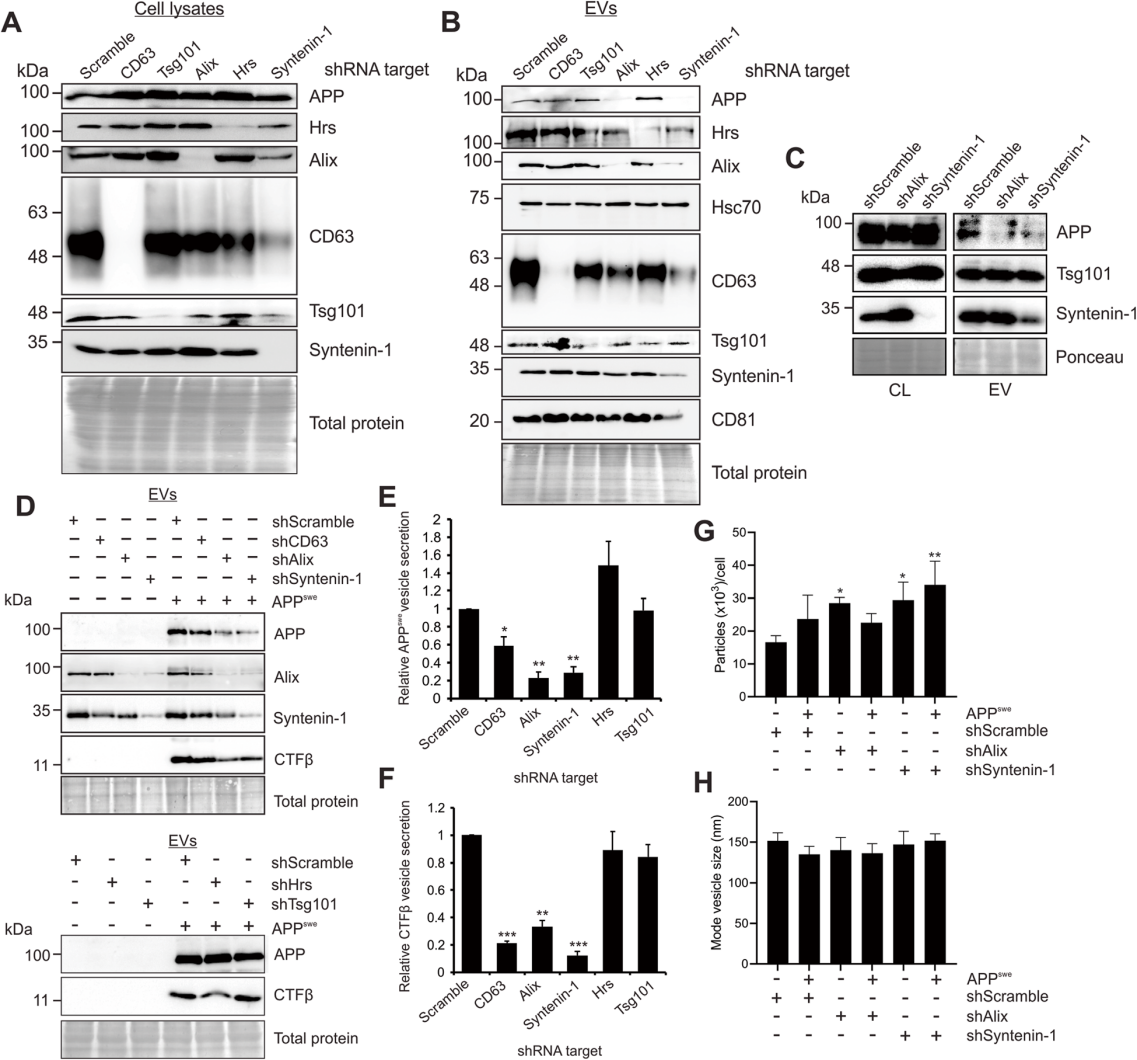
Alix and Syntenin-1 drive mutant APP packaging into secreted vesicles. **a** Immunoblot analysis of cell lysates following activation of stably transduced inducible shRNA gene knockdowns targeting ESCRT proteins Hrs (shHrs) and Tsg101 (shTsg101), tetraspanin protein CD63 (shCD63), and Alix (shAlix) and Syntenin-1 (shSyntenin-1), and subsequent transfection of APP^swe^. Equal protein mass was loaded into gels. **b** Immunoblots of corresponding secreted EVs, with equal volume loaded. **c**) Comparison of endogenous APP secretion into EVs following Alix or Syntenin-1 knockdown. **d**) Immunoblot analysis of CTFβ peptides secreted into EVs. Quantitation of (**e**) APP and (**f**) CTFβ secreted into vesicles from shRNA-expressing cells, normalized to cellular levels. Blots are representative images from four to five repeated independent experiments. Statistical differences were determined by two-way student’s t-test. Nanoparticle tracking analysis showing (**g**) quantity and (**h**) size of small EVs from control, shAlix, and shSyntenin-1 cells with or without APP^swe^ expression. One-way ANOVA was used to determine statistical significance across samples from three independent experiments. ***, *p* < 0.001; **, *p* < 0.01; *, *p* < 0.05

Various ESCRT- dependent or -independent pathways are involved in trafficking and secretion of EV cargo. In this study, we utilized a series of gene knockdowns to elucidate the major mechanism of APP sorting into small EVs. Efficient knockdowns of ESCRT pathway proteins Hrs and Tsg101 were achieved, in addition to knockdowns of the ESCRT-associated protein Alix, tetraspanin protein CD63, and Syntenin-1 (Fig. 2a). Strikingly, in cells expressing APP^swe^, the mutant precursor protein was packaged into EVs despite considerable loss of Hrs, Tsg101, and CD63 protein expression (Fig. 2b). However, efficient APP packaging into small EVs appeared to be dependent upon the presence of Alix and Syntenin-1 (Fig. 2b and d). Endogenous APP secretion was likewise diminished with Alix and Syntenin-1 knockdown (Fig. 2c). Interestingly, knockdown of Alix decreased the amount of Syntenin-1 secreted into vesicles, and a similar effect was seen in Alix release following Syntenin-1 knockdown (Fig. 2b), supporting a common pathway of EV sorting including these two proteins. We note that the relatively decreased quantity of APP secreted into EVs following CD63 knockdown may have been primarily due to increased cellular levels of APP, the mechanism of which is not understood, but could be due to intracellular trafficking or degradation variation in the setting of CD63 protein depletion.

As expected, untransfected cells expressing endogenous APP demonstrated very little secretion of APP or CTFβ into EVs (Fig. 2d). However, higher levels of CTFβ derived from APP^swe^ mutant cells were secreted in association with vesicles, and similarly dependent on Alix and Syntenin-1 for packaging (Fig. 2d and f). Interestingly, decreased secretion of APP and CTFβ was not a factor of overall diminished EV production. In fact, depletion of Alix and Syntenin-1 appeared to increase total vesicle production even slightly (Fig. 2g), though the source of these EVs remains unknown. No significant difference in vesicle size was seen following Alix or Syntenin-1 knockdown (Fig. 2h).

**Fig. 3:**
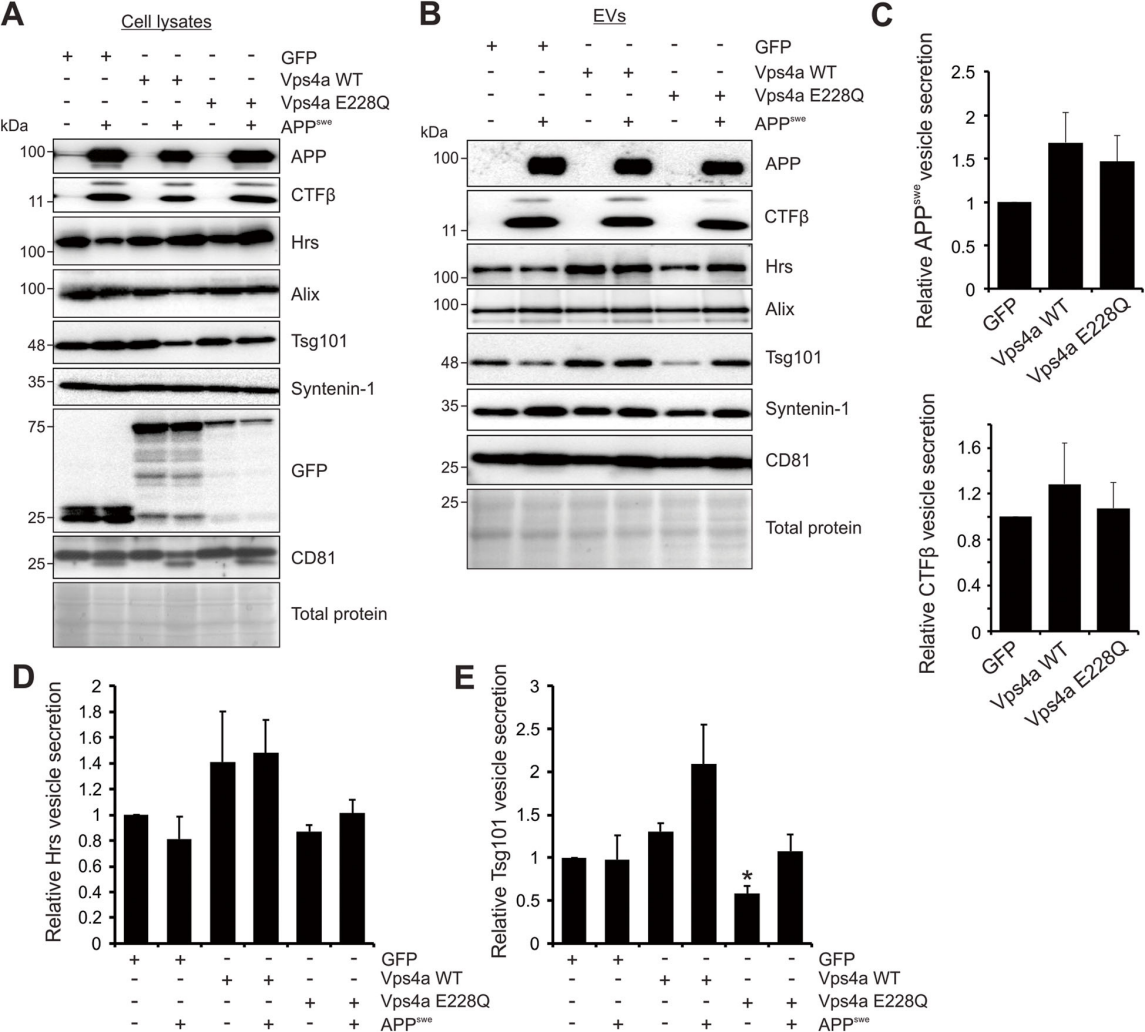
APP is secreted into vesicles independent of ESCRT machinery. **a** GFP-tagged wild-type Vps4a and corresponding dominant negative construct (E228Q) were transfected into HEK293 cells expressing APP^swe^. Upper bands in CTFβ blots likely represent protein dimers due to a molecular weight of approximately 20 kDa and recognition by the same specific antibody. **b** Immunoblot analysis of EVs secreted from Vps4a WT and dominant negative transfected cells. Blots are representative images from three to four repeated independent experiments. Resultant quantitation of (**c**) vesicle APP^swe^ and CTFβ, (**d**) Hrs, and (**e**) Tsg101 vesicle secretion, normalized to cellular levels. One-way ANOVA was used to determine statistical significance across samples from three independent experiments. *, *p <* 0.05

It has been reported that overexpression of ESCRT-II proteins may rescue depletions of earlier ESCRT components [30, 31]. Therefore, it was considered possible that APP release was rescued by a downstream ESCRT compensatory pathway, rather than packaged into vesicles entirely independent of early ESCRT proteins. To address this question, we introduced a GFP-tagged dominant negative mutant of Vps4a (Vps4a E228Q) [44], an AAA ATPase that plays an essential role in ESCRT complex disassembly, membrane deformation, and fission events crucial for the final stages of intraluminal vesicle formation (Fig. 3a). Vesicle secretion of early ESCRT proteins Hrs and Tsg101 appeared mildly increased following overexpression of wild-type Vps4a (Fig. 3b, d, e). However, in the presence of the dominant negative Vps4a protein, Hrs and Tsg101 secretion was reduced or returned to baseline. Notably, a decrease in APP or CTFβ vesicle packaging was not observed in cells expressing the dominant negative Vps4a protein (Fig. 3b-c), affirming the suspected ESCRT-independent sorting pathway.

**Fig. 4:**
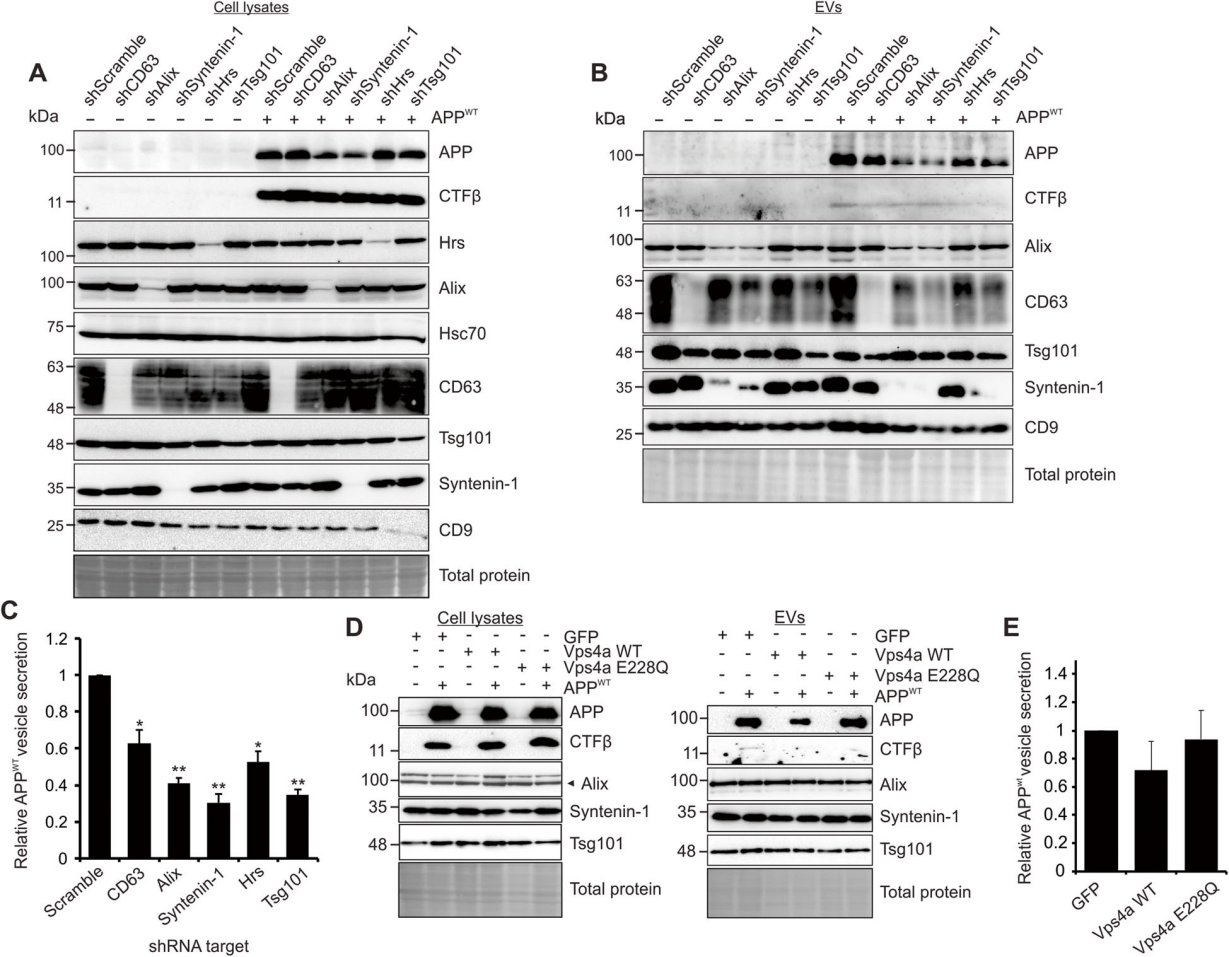
Wild-type APP is similarly dependent upon Alix and Syntenin-1 for secretion. **a** Inducible shRNA knockdown cells were transfected with wild-type APP (APP^WT^), equal mass loaded. **b** EVs were harvested from APP^WT^ transfected cells for immunoblot analysis, equal volume loaded. **c** Quantitation of APP^WT^ secreted into EVs from knockdown cells, normalized to cellular levels. **d** GFP-tagged wild-type Vps4a and corresponding dominant negative construct (Vps4a E228Q) were transfected into HEK293 cells expressing APP^WT^. EVs were collected and analyzed by immunoblot analysis, equal volume loaded. **e** Quantitation of APP^WT^ secreted into EVs from wild-type Vps4a and dominant negative Vps4a transfected cells. Blots are representative images from three to five repeated independent experiments. T-test: *, *p <* 0.05; **, *p <* 0.01

To further determine whether the Alix and Syntenin-1 reliant sorting of APP was specific to the mutant protein, human wild-type APP was introduced into cells carrying the genetic knockdowns (Fig. 4a). Of note, high levels of transfected wild-type APP were sufficient to produce detectable levels of the catabolite CTFβ in cells, although CTFβ derived from APP^WT^ was not efficiently secreted into EVs. In these experiments, secretion of wild-type APP was similarly diminished following Alix, Syntenin-1 knockdown (Fig. 4b-c). Again, CD63 knockdown slightly reduced vesicle APP^WT^ secretion, while a mild increase in cellular levels of APP were noted. Interestingly, knockdown of earlier ESCRT proteins Hrs and Tsg101 also reduced APP^WT^ vesicle packaging. Again, inhibition of Vps4a using the dominant negative protein Vps4a E228Q demonstrated no significant impact on APP or Aβ packaging (Fig. 4d-e). These findings suggest APP is normally sorted into EVs through both ESCRT and Alix-Syntenin-1 pathways, but the mutant protein may rely more heavily on the Alix-Syntenin-1 pathway or can escape earlier ESCRT protein knockdowns.

**Fig. 5:**
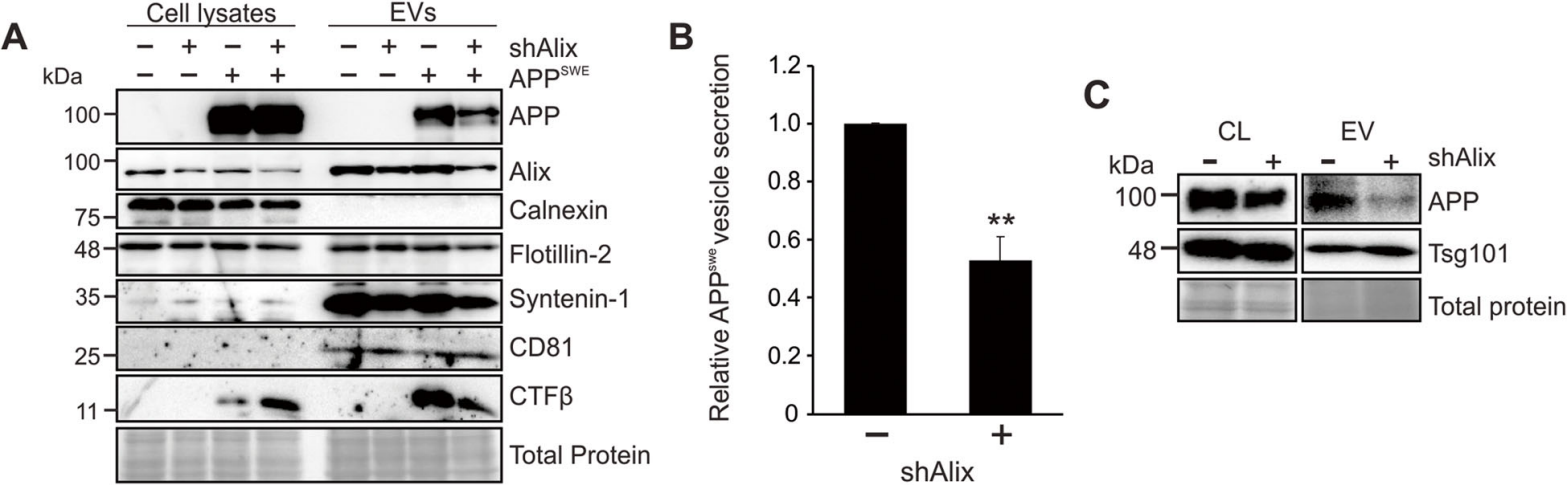
Alix-dependent APP trafficking is conserved in neuron-like cells. **a** SH-SY5Y neuroblastoma cells stably transduced with inducible shRNA targeting Alix were terminally differentiated into neuron-like cells, then transfected with APP^swe^. Cell lysates and cell-derived EVs were harvested for immunoblot analysis. **b** Quantitation of APP^swe^ secretion into vesicles. **c** Representative immunoblots of cellular levels and vesicular secretion of endogenous SH-SY5Y APP. Blots are representative images from two to three repeated independent experiments. T-test: **, *p <* 0.01

Elucidation of the mechanisms surrounding vesicular trafficking of APP and its catabolites is perhaps most relevant in the context of neuronal EV secretion. To examine whether this apparent mechanism of APP sorting was conserved, human neuroblastoma (SH-SY5Y) cells containing inducible Alix shRNA were differentiated into mature neurons. Decreased vesicle secretion of both Alix and Syntenin-1 was seen following Alix knockdown (Fig. 5a). Similar to the results observed in HEK293 cells, APP and CTFβ secretion was also significantly reduced in these cells, suggesting a comparable mechanism of APP trafficking and vesicle secretion (Fig. 5b). Interestingly, intracellular CTFβ levels appeared increased following Alix depletion. It is possible that impaired trafficking of the precursor protein leads to differential amyloidogenic processing, or perhaps degradation mechanisms are unable to compensate for the impaired secretion. Finally, evaluation of endogenous APP secretion demonstrated similar dependency on Alix in SH-SY5Y cells (Fig. 5c).

### Intracellular localization of APP is dependent upon Alix and Syntenin-1

**Fig. 6:**
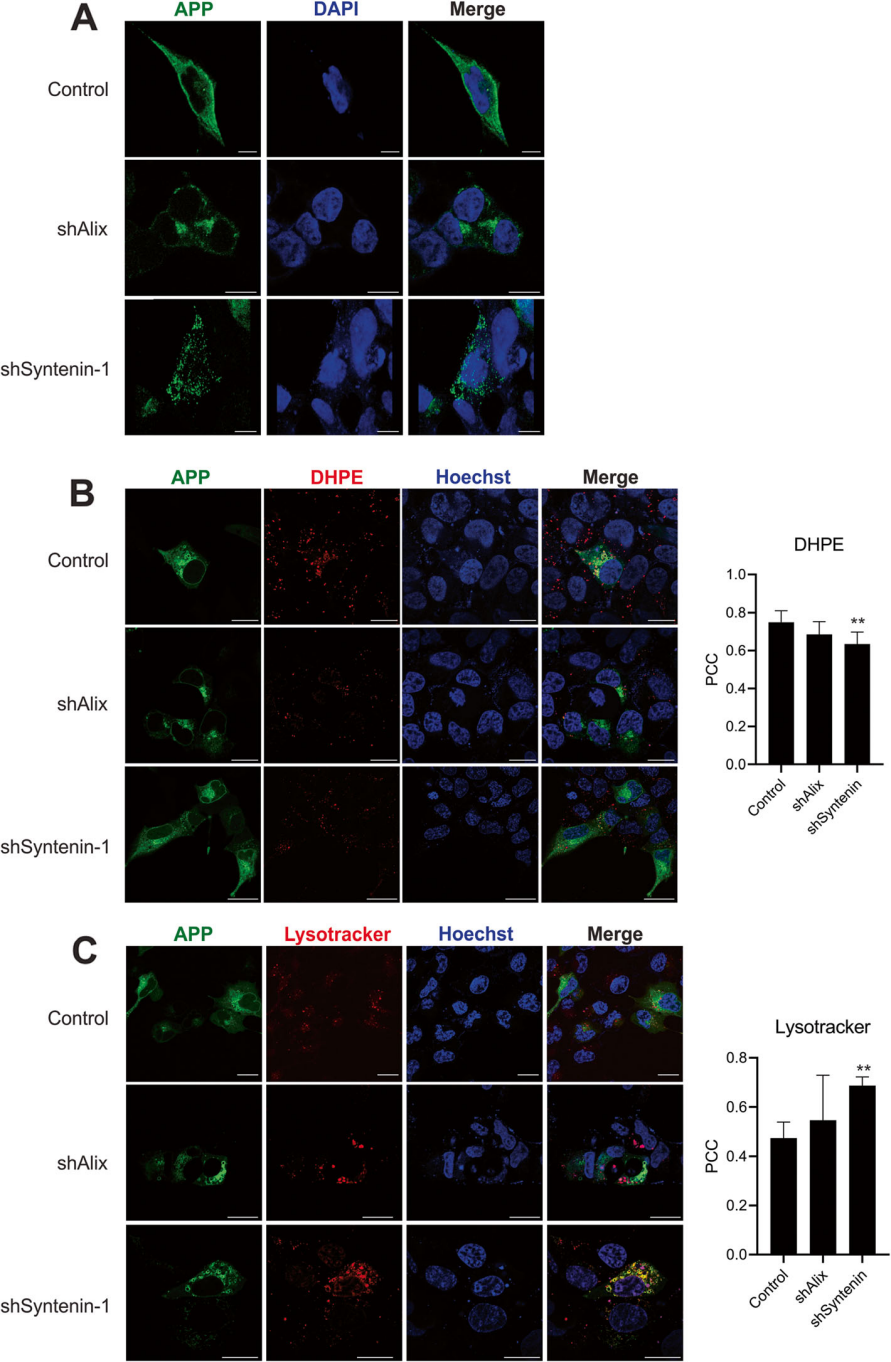
APP endolysosomal localization is altered in the absence of Alix and Syntenin-1. **a** APP immunofluorescent staining was performed on control HEK293 cells expressing APP^swe^ or following induction of Alix (shAlix) or Syntenin-1 (shSyntenin-1) shRNA knockdown. To assess subcellular compartment localization of APP^swe^ in the absence of Alix and Syntenin, GFP-tagged APP^swe^ was introduced into cells. Cells were stained with (**b**) DHPE or (**c**) Lysotracker before live-cell imaging on a Zeiss LSM 880 confocal microscope. Images are representative across at least three independent experiments. Scale bar = 20 μm. Pearson’s Correlation Coefficient (PCC) calculated from ≥10 cells. One-way ANOVA: **, *p <* 0.01. *, *p <* 0.05

**Fig. 7:**
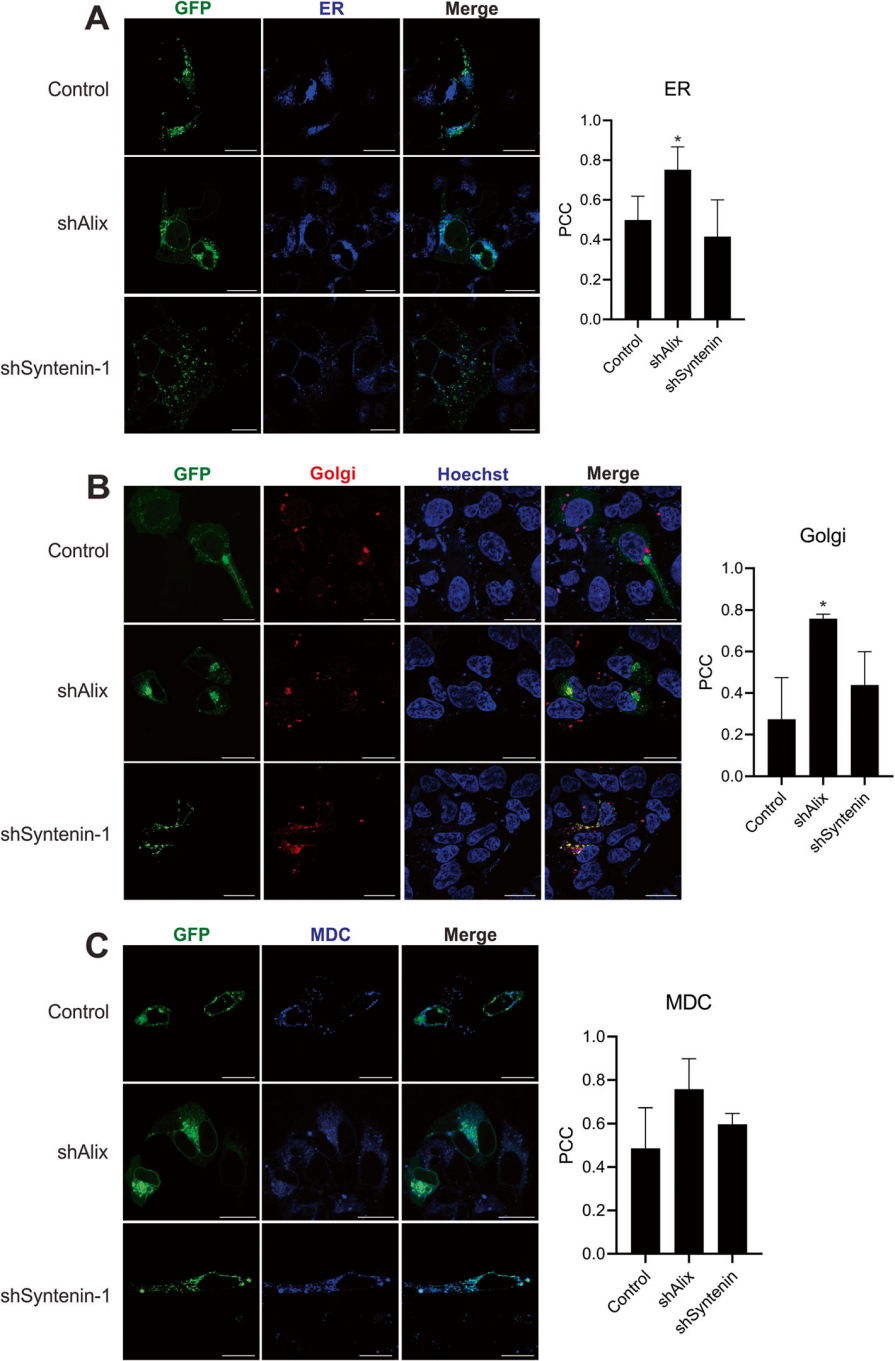
APP endoplasmic reticulum  (ER) and Golgi localization is altered in the absence of Alix and Syntenin-1. Cells expressing GFP-tagged APP^swe^ were stained with an (**a**) Blue-white fluorescent ER marker, (**b**) RFP-tagged Golgi network marker, or (**c**) monodansylcadaverine (MDC) before live-cell imaging on a Zeiss LSM 880 confocal microscope. Images are representative across at least three independent experiments. Scale bar = 20 μm. Pearson’s Correlation Coefficient (PCC) calculated from ≥10 cells. One-way ANOVA: **, *p <* 0.01; *, *p <* 0.05

To determine whether intracellular localization of the APP^swe^ protein was altered with Alix and Syntenin-1 knockdowns, cellular APP immunostaining was performed and imaged by confocal microscopy (Fig. 6a). APP^swe^ localized to several compartments in control cells, with prominent localization to the plasma membrane and perinuclear region. In contrast, Alix knockdown produced a distinct phenotype where APP was concentrated much more in the perinuclear area, while Syntenin-1 knockdown cells demonstrated a punctate localization of APP. To further characterize subcellular compartment localization of the amyloid precursor protein, a GFP-tagged APP^swe^ construct was expressed in control cells or those containing doxycycline-inducible Alix or Syntenin-1 shRNA. In control cells, APP localized to several subcellular compartments including 1,2-Dihexadecanoyl-sn-Glycero-3-Phosphoethanolamine (DHPE)-positive endosomes, lysosomes, the endoplasmic reticulum (ER), and Golgi network (Fig. 6b-c, 7a-b). These findings are consistent with the known trafficking route of the protein. In contrast, following Alix knockdown, APP primarily localized to the ER and Golgi (Fig. 7a-b), while endolyososomal localization of the protein was unchanged (Fig. 6b-c), and plasma membrane localization was attenuated. Finally, in Syntenin-1 shRNA expressing cells, APP was enriched in the lysosomal compartments (Fig. 6c), and decreased in DHPE-positive endosomes (Fig. 6b). No significant change in MDC-positive autophagic vacuole localization of APP was seen across cell groups. These alterations noted in APP trafficking within cells likely explain the reductions in vesicle secretion of the precursor protein and CTFβ in the absence of Alix and Syntenin-1.

### Knockdown of Alix and Syntenin-1 ameliorates the neurotoxic effect of APP

**Fig. 8:**
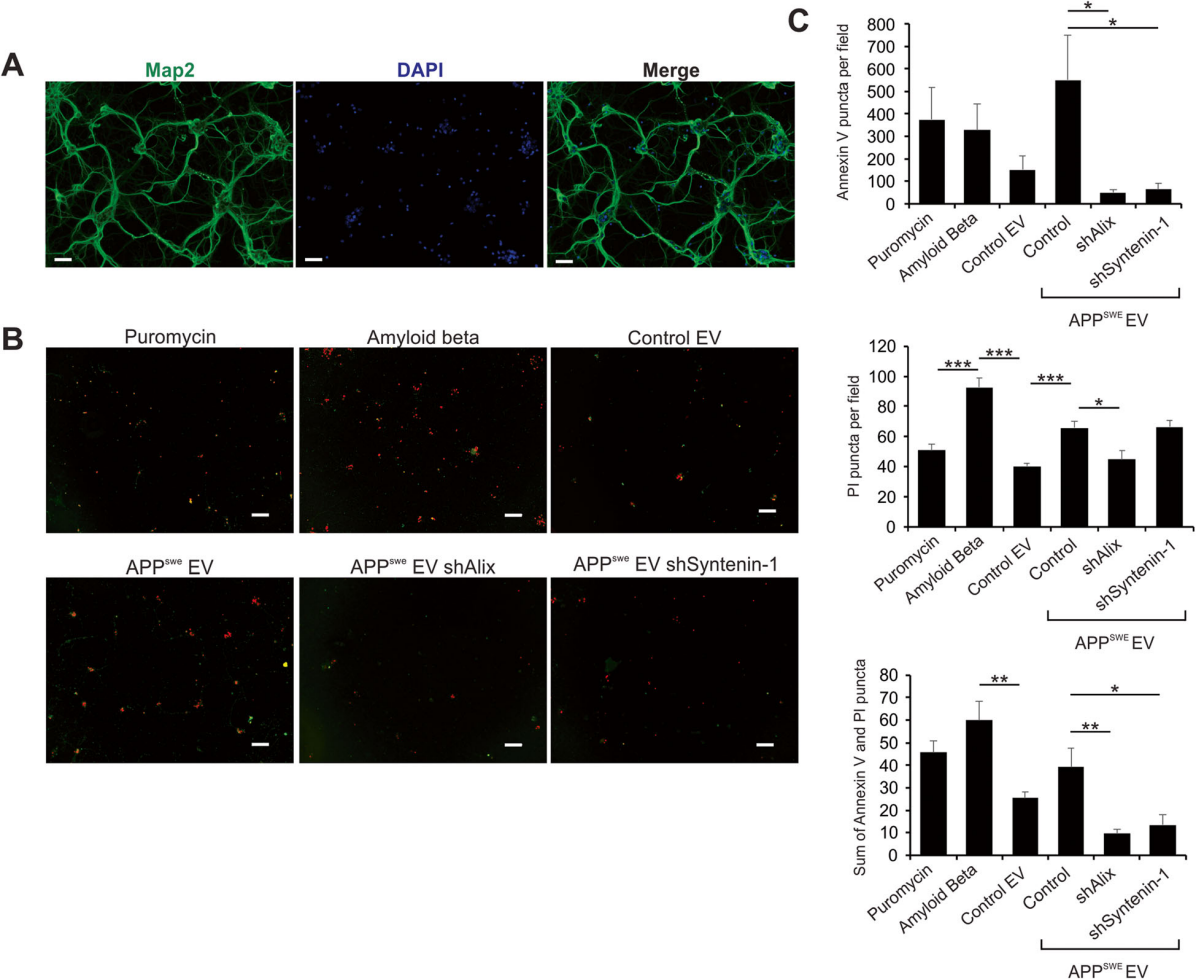
Alix and Syntenin-1 knockdown ameliorates the neurotoxicity conferred by APP^swe^-containing EVs. **a** Differentiated primary neurons were stained with anti-Map2 to demonstrate neuronal origin. Scale bar = 50 μm. **b** Neurons were treated with puromycin, purified oligomeric amyloid beta peptide, or EVs derived from control, Alix shRNA, or Syntenin-1 shRNA cell lines expressing APP^Swe^. Neuronal cells were stained with both Annexin V (green) and propidium iodide (PI; red) to assess for levels of apoptosis and cell necrosis, respectively. Scale bar = 100 μm. **c** Cell death was quantitated by Annexin V and PI puncta per microscopic field. A minimum of 200 cells were counted per group. Imaging was performed using a Keyence BZ-X710 fluorescence microscope. Images are representative across three independent experiments. One-way ANOVA with post hoc Tukey’s multiple comparison test: ***, *p <* 0.001; **, *p <* 0.01; *, *p <* 0.05

Finally, the neurotoxic effects of APP-containing EVs were compared to those of EVs following Alix and Syntenin-1 knockdown. Primary neuronal cells were prepared from the cerebral cortex of day 0 mice and cultured in vitro. Cells were stained with anti-Map2 to demonstrate neuronal identity (Fig. 8a). Following treatment with puromycin, purified oligomerized amyloid beta, or cell-derived EVs, differentiated neuronal cells were stained with Annexin V (green) and propidium iodide (PI; red) to assess levels of apoptosis and cellular necrosis, respectively (Fig. 8b). EVs secreted from control cells conferred relatively low levels of neurotoxicity, while transfer of APP^swe^-containing vesicles resulted in cell death levels comparable to purified Aβ peptide and puromycin-treated cells (Fig. 8b-c). This EV-mediated neurotoxic effect was significantly diminished following Alix or Syntenin-1 knockdown. Interestingly, necrotic cell death appeared to predominate in the setting of purified amyloid beta peptide, while EVs mediated mostly apoptotic cell death.

**Fig. 9:**
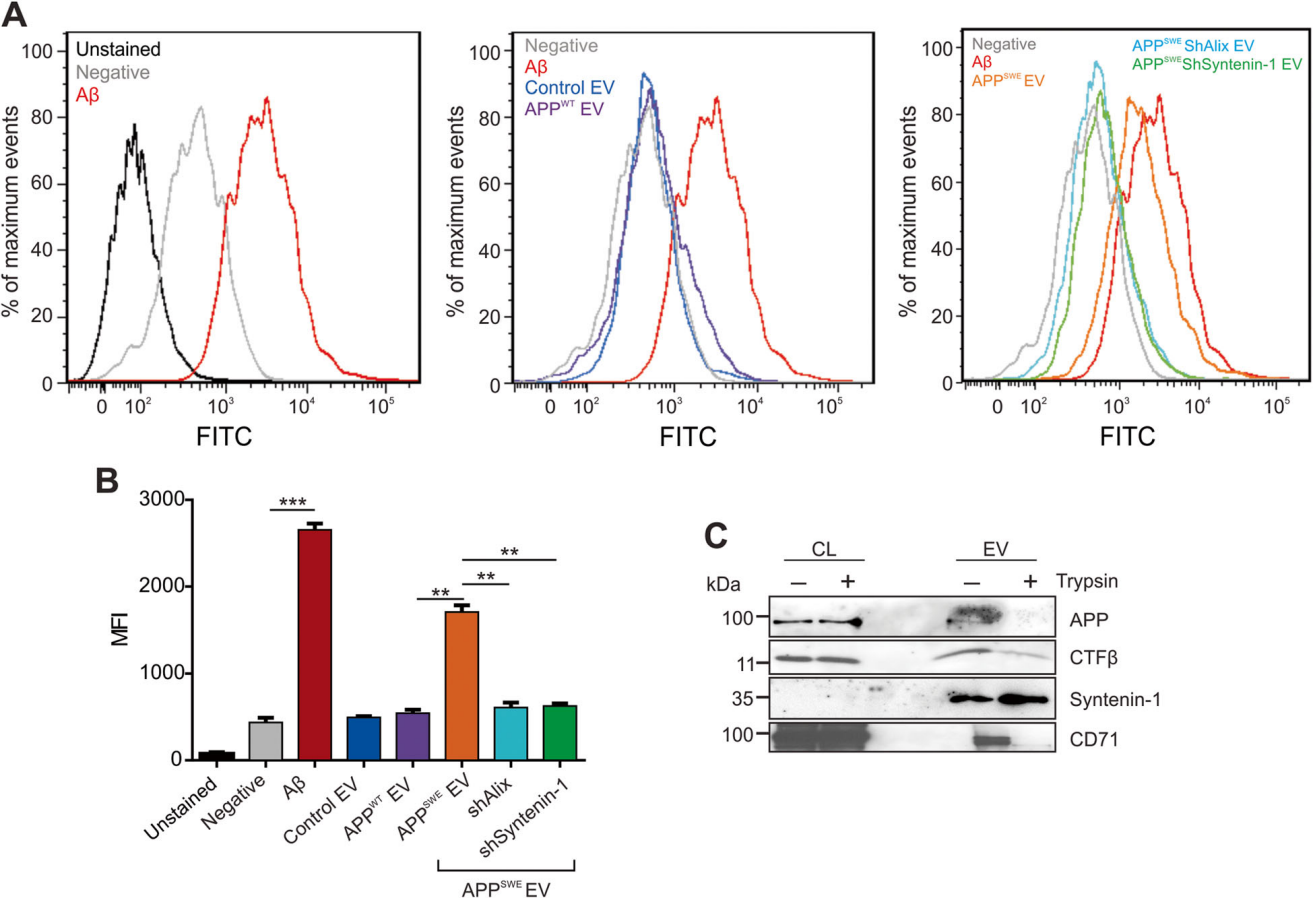
Vesicular amyloid-induced reactive oxygen species are decreased following Alix and Syntenin-1 depletion. **a** Reactive oxygen species detected by flow cytometry following purified amyloid beta peptide or EV treatment. **b** Quantitation of reactive oxygen species by flow cytometry analysis over independent experiments. **c** Western blot analysis of EVs pre-treated with trypsin before ultracentrifugation wash and lysis. Blots are representative images from three independent experiments. MFI = mean fluorescence intensity. One-way ANOVA with post hoc Tukey’s multiple comparison test: ***, *p <* 0.001; **, *p <* 0.01

Previous research has focused on the contribution of increased reactive oxygen species (ROS) formed within cells during the progression of AD [45]. To assess the impact of EV treatment on ROS formation, human induced pluripotent stem (iPSK3) cells were differentiated into cortical neurons through spheroid formation in suspension. A flow cytometry-based assay was used to measure ROS production in spheroid cultures following treatment with purified Aβ peptide or EVs derived from control HEK293 cells, cells expressing APP^WT^, or cells expressing APP^swe^ in addition to shRNA targeting Alix or Syntenin-1. EVs containing APP^swe^ secreted from control cells conferred high levels of ROS, near comparable to spheroids treated with amyloid beta peptide (Fig. 9a-b). Depletion of Alix and Syntenin-1 significantly mitigated this effect on ROS production. These findings expectedly account for, at least in part, the decrease in overall neurotoxicity seen following the transfer of similar EVs, likely a factor of decreased mutant APP and CTFβ, and perhaps low levels of Aβ, in vesicles following Alix or Syntnein-1 knockdown.

It is believed that the neurotoxicity elicited by Aβ is mediated by extracellular binding of the peptide to putative Aβ-binding receptors on the neuronal cell surface [46]. To determine whether APP and CTFβ are present on the surface of isolated EVs and thereby able to mediate an analogous effect, EVs were treated with trypsin to cleave external protein epitopes. Following trypsin treatment and ultracentrifugation wash, immunoblot analysis was performed on lysed EV protein (Fig. 9c). Levels of detectable APP and CTFβ were decreased in addition to transmembrane protein CD71. In contrast, intravesicular levels of Syntenin-1 remained unchanged. Therefore, it is likely that the cleaved APP products remain predominately associated with the external surface of vesicles, mediating their neurotoxic effects.

## Discussion

The contribution of amyloid protein deposition to pathologic neurodegeneration has been a traditional and durable hypothesis with regards to the progression of Alzheimer’s disease. Accruing evidence has pointed to the roles of extracellular vesicles in the secretion and uptake of misfolded proteins, including amyloid, synuclein, and prion proteins [20, 21, 23, 47–52]. It is noted that only a small proportion of secreted Aβ is likely associated with EVs [23, 53], as similarly concluded in this study. Given the transmembrane nature of the precursor protein and its C-terminal fragment, the enrichment of circulating APP and CTFβ in EV membranes is expected, though not demonstrated in this study.

The net effect of EV secretion of amyloid precursor protein and its neurotoxic catabolites is not entirely clear, however. Several studies have proposed that Aβ-carrying vesicles may contribute to the propagation of amyloid secretion and plaque formation in the brain [27, 54], while apparently contradictory data suggest that neuronal EVs may aid in clearance of toxic proteins by microglia uptake [55] or by enzymatic degradation of amyloid beta [56]. Given the abundance of data describing the immunoregulatory effects of EVs, it is also possible that secreted vesicles contribute in part to the inflammatory response seen in AD. As others have alluded to [57], amyloid-containing vesicles may very well impart elements of both cellular protection and toxicity within the complexities of the neurodegenerative disease, aiding in clearance and sequestering (plaque development) of soluble amyloid from one neuron at the expense of uptake in another.

The C-terminal fragment of APP has been shown to be associated with amyloid plaques, and previous research has shown it accumulates in the brain of various mouse models [28]. Aggregation of CTFβ occurs mainly in endolysosomal compartments and may contribute to lysosomal dysfunction, one of the hallmarks of AD pathology [24, 25, 58, 59]. Similar to Aβ, oligomerization of CTFβ can occur, and oligomers may be detected in secreted EVs [28]. The role of CTFβ in AD pathology could also explain some of the adverse side effects of γ-secretase inhibitor therapies for AD [24]. Of course, while we demonstrate that the predominant APP metabolite in EVs secreted from APP^swe^ transfected cells is likely CTFβ, we do not rule out the possibility of a lower level of Aβ or CTFα packaging.

In order to better understand the roles of pathogenic proteins in AD progression, an increased understanding of the mechanisms of intracellular and intercellular trafficking of these target proteins is necessary. Here, we demonstrate that a mutant amyloid precursor protein (APP^swe^) is trafficked into small EVs for secretion by an Alix-Syntenin-1 dependent mechanism, independent of early or late ESCRT machinery. It is likely that CD63 also engages in this ESCRT-independent pathway, as previously demonstrated [34]. This pathway appears to be conserved in terminally differentiated neuronal cells. Although this study is practically limited by the need to express high levels of exogenous APP, as vesicle-associated CTFβ is difficult to detect under endogenous conditions, a demonstrable decrease in endogenous SY5Y and HEK293 cell-derived EV APP was similarly observed. Of note, hampered EV production from cells lacking Alix and Syntenin-1 was not seen, and instead, detectable increases in vesicles were noted to be secreted from these cells. These findings suggest that the Alix-Syntenin-1 pathway may be more involved in protein trafficking and cargo loading into vesicles, rather than EV budding or primary biogenesis, as has been previously suggested. It is possible that the augmented vesicle production may be compensatory in the setting of disrupted cargo packaging into individual EVs [29, 60].

Interestingly, while Alix and Syntenin-1 are necessary for proper intracellular trafficking of APP, early ESCRT proteins Hrs and Tsg101 appear to be additionally helpful in regulating the release of vesicular wild-type full length APP in this study. The discordance of early ESCRT involvement  between wild-type and mutant APP trafficking is not completely clear. It is possible that APP^WT^ requires ESCRT recognition of its ubiquitination status to guide sorting from the plasma membrane into endosomes, while APP^swe^ bypasses this limitation. Future studies are required to elicit a better understanding of this mechanism [61–63].

Altered intracellular trafficking of APP clearly influences the secretion of the full-length and processed protein. It is possible that depletion of key proteins involved in vesicle biogenesis, including Alix and Syntenin-1, may lead to general disruption of endosomal compartment organization. However, decreased secretion of APP was seen following Alix and Syntenin-1 knockdowns in the absence of global vesicle reduction, suggesting a more specific interaction guiding intracellular APP trafficking. In the absence of Alix, APP was observed to be sequestered in perinuclear regions of the cell, primarily localized in the endoplasmic reticulum and Golgi. It has previously been proposed that Syntenin-1 binds syndecan assemblies on the surface of endosomal membranes; this complex subsequently recruits Alix to initiate processes involved in vesicle budding for ILV formation. The proposed order of these protein interactions is difficult to determine in the context of APP localization, as seen in this study. While the mechanism and significance of APP sequestration in the ER and Golgi is unclear, it is possible that this may in part reflect the importance of Alix-mediated formation of Golgi transport vesicles that bud from the endoplasmic reticulum, as previously described [64]. In this case, following Alix depletion, immature APP trafficking from the ER and Golgi may be limited. In contrast, APP localized predominately to lysosomes following Syntenin-1 knockdown. In the absence of the syndecan adaptor protein, Alix and Syntenin-1-mediated ILV formation is likely hampered, and APP is likely diverted to lysosomal compartments in lieu of incorporation into endosomal ILVs and extracellular vesicle secretion. Additional work will be necessary to unfold the protein-protein interactions driving these cellular pathways toward EV secretion.

Despite the unknown surrounding APP intracellular trafficking in the absence of Alix and Syntenin-1, the impact of these protein depletions on amyloid-mediated neurotoxicity was particularly pronounced in this study. Consistent with the reduction in APP and CTFβ secreted into EVs, the neurotoxic effect (by cell viability and ROS production assays) conferred by vesicles was similarly decreased following genetic knockdown of Alix and Syntenin-1. In comparison, transfer of APP^swe^-containing vesicles induced neuronal cell death comparable to amyloid beta peptide alone, suggesting that cleaved APP on the external surface of EVs is present in an active or accessible form, as has been suggested [53]. It is also possible that the neurotoxic effect may be in part mediated by non-APP related factors not examined in this study.

To our knowledge, ROS induction by CTFβ has not been well described. Here, we propose that EV-associated CTFβ causes a ROS response similarly to Aβ peptide. Of note, we observed remnant detectable CTFβ following trypsin digestion of EV membrane proteins in comparison to near fully digested APP and CD71. It is plausible that this reflects relative protease resistance of CTFβ, perhaps due to oligomerization on the surface of EVs, which is reduced on subsequent gel analysis. Previous evidence suggests that amyloid-induced neurotoxicity is mediated by binding of the peptide to amyloid receptors on the surface of recipient cells [46]. In this study, we demonstrate the predominant topographical distribution of both APP and CTFβ to the membrane or external surface of EVs, further supporting the ability of vesicles to carry these biologically active proteins. We also note that apoptotic cell death appears to dominate as the major mechanism of neuronal death conferred by amyloid carrying EVs while purified Aβ peptide induces necrosis predominately. It is possible that CTFβ mediates a separate neurotoxic effect compared to Aβ that could be a result of its partial localization to mitochondria-associated ER membranes leading to mitochrondrial dysfunction [61]. Further work will be necessary to understand the mechanisms underlying these pathways.

## Conclusions

A previous study by Dinkins et al. demonstrated that reduction of EVs in vivo by inhibition of ceramide production led to decreased amyloid plaque deposition and amelioration of the neurocognitive effects seen in an AD mouse model expressing a similar mutant APP [27]. Our present in vitro work supports the proposed neurotoxicity mediated by EVs containing mutant APP and CTFβ, and further elucidates a mechanism of APP trafficking into small EVs for secretion. Here we use a targeted genetic approach to knockdown key proteins involved in APP intracellular trafficking, implicating the recently described Alix-Syntenin-1 pathway in APP sorting and vesicular secretion. Sphingomyelinase inhibitors have rather been shown to exhibit a global reduction of EV populations released from cells, the impact of which is unknown, particularly in the context of in vivo mouse studies. Our findings in this study provide tools to further, and more specifically, examine the impact of reducing APP vesicle secretion on the neurodegeneration and cognitive dysfunction seen in AD mouse models. These data may further shed light on the broader protein-protein interactions that guide vesicular protein trafficking and EV secretion, an area of interest that remains rather elusive across many fields of ongoing scientific research.

## Methods

### Cell culture

HEK293 (ATCC; CRL-1573) were cultured in Dulbecco modified Eagle medium (DMEM; Sigma; D5796). SH-SY5Y cells (a gift from Cao Chuanhai, University of South Florida) were cultured in DMEM/ Ham’s F-12 50/50 mix (DMEM/ F-12 50/50; Corning; 10–090-CV). Medium was supplemented with 10% fetal bovine serum (FBS; Seradigm; 1400–500), 2 mM L-glutamine (Corning; 25–005-CI), 100 IU of penicillin-streptomycin (Corning; 30–002-CI), and 100 μg/mL:0.25 μg/mL antibiotic/antimycotic (Corning; 30–004-CI). Serum used for experiments was depleted of extracellular vesicles by ultracentrifugation at 100,000×g for 20 h and filtered through a 0.2-μm filter prior to being added to culture medium. At the time of harvest, live cells were counted with an automated cell counter (Cellometer Vision, software version 2.1.4.2; Nexcelom Biosciences) by staining with 0.2% trypan blue (Sigma; T8154) in phosphate-buffered saline (PBS). Total live cell counts were used to derive particles per cell in subsequent nanoparticle tracking analyses.

### Primary cortical neuron preparation

All animal procedures were carried out in accordance with the guidelines for the Florida State University Institutional Animal Care and Use Committee (ACUC) and all studies were performed in accordance with the recommendations in the National Institute of Health’s Guide for the Care and Use of Laboratory Animals. Cultures of primary cortical neurons were prepared using a standard procedure. Briefly, cerebral cortices were dissected from postnatal day 0 mice with the aid of a stereo microscope. Isolated cerebral cortices were digested with papain (Worthington Biochemical Corporation; LS003119) for 5 min at 37 °C. Isolated neurons were seeded on poly-D-lysine-coated plates at a density of 470 cells/mm^2^ and were cultured in neurobasal A medium (Gibco; A3653401) supplemented with B-27 (Gibco; A3582801), 0.5 mM L-glutamine, and penicillin-streptomycin at 37 °C and 5% CO_2_.

### Generation of shRNA constructs

Oligonucleotides targeting ALIX, SDCBP (encoding Syntenin-1), TSG101, HRS, and CD63 genes were constructed using target sequences obtained from the Broad Institute GPP Web Portal. The control scramble sequence was generated using the Invivogen shRNA scramble tool as previously described [65]. The oligonucleotides listed below were annealed and ligated using T4 ligase between the Bgl II/Hind III sites of pENTR/pTER+ (Addgene plasmid # 17453, a gift from Eric Campeau & Paul Kaufman). Entry clones were then sequenced for verification, and recombined with the destination vector pLenti X1 Zeo Dest (Addgene plasmid # 17299, a gift from Eric Campeau & Paul Kaufman) using LR recombination (Invitrogen #11791–020) according to the manufacturer’s instructions, and as previously described [65].

ALIX:

GATCCCGCATAATCAAGGCACTGTAAAGTGTGCTGTCCTTTACAGTGCCTTGATTATGCTTTTTGGAAA and AGCTTTTCCAAAAAGCATAATCAAGGCACTGTAAAGGACAGCACACTTTACAGTGCCTTGATTATGCGG.

SDCBP (Syntenin-1): GATCCCTATAGCATACTTGCATCTTTAGTGTGCTGTCCTAAAGATGCAAGTATGCTATATTTTTGGAAA and AGCTTTTCCAAAAATATAGCATACTTGCATCTTTAGGACAGCACACAAAGATGCAAGTATGCTATAGG.

TSG101:

GATCCCGTACGTCTTCTGTCCCGTAAAGTGTGCTGTCCTTTACGGGACAGAAGACGTACTTTTTGGAAA and.

AGCTTTTCCAAAAAGTACGTCTTCTGTCCCGTAAAGGACAGCACACTTTACGGGACAGAAGACGTACGG.

CD63 (combined for lentivirus production):

GATCCCCAACGAGAAGGCGATCCATAAGTGTGCTGTCCTTATGGATCGCCTTCTCGTTGTCTTTTTGGAAA and.

AGCTTTTCCAAAAACAACGAGAAGGCGATCCATAAGGACAGCACACTTATGGATCGCCTTCTCGTTGGG.

GATCCCTGGGATTAATTTCAACGAGAAGTGTGCTGTCC TTCTCGTTGAAATTAATCCCATTTTTGGAAA and.

AGCTTTTCCAAAAATGGGATTAATTTCAACGAGAAGGACAGCACACTTCTCGTTGAAATTAATCCCAGG.

HRS:

GATCCCACGGTATCTCAACCGGAACTAGTGTGCTGTCCTAGTTCCGGTTGAGATACCGTCTTTTTGGAAA and.

AGCTTTTCCAAAAAACGGTATCTCAACCGGAACTAGGACAGCACACTAGTTCCGGTTGAGATACCGTGG.

Scramble:

GATCCCGAGCTTCGCGATCCAAGATAAGTGTGCTGTCCTTATCTTGGATCGCGAAGCTCTTTTTGGAAA and AGCTTTTCCAAAAAGAGCTTCGCGATCCAAGATAAGGACAGCACACTTATCTTGGATCGCGAAGCTCGG.

### Lentivirus production and stable cell generation

Lentivirus particles for transduction and stable cell generation were produced in HEK293T cells following lipofectamine transfection of expression plasmids (pLenti CMV TetR BLAST and plenti X1 shRNA plasmids) and packaging plasmids pMD2.G (Addgene; number 12259; a gift from Didier Trono) and PSPAX2 (Addgene; number 12260; a gift from Didier Trono) according to the manufacturer’s instructions (Invitrogen, L3000015). Of note, to increase efficiency of the knockdown, lentivirus particles for shRNA constructs targeting CD63 were generated by mixing two expression plasmids, generated by the dual oligonucleotides listed. Medium was collected at 48, 72, and 96 h post-transfection, centrifuged for 10 min at 1000×g, filtered through a 0.45-μm filter, and frozen at − 80 °C until use.

Cells stably expressing shRNA of different genes under the control of a tetracycline-inducible promoter were created by first transducing HEK293 or SH-SY5Y cells with lentivirus particles containing pLenti CMV TetR BLAST (Addgene; number 17492). Stable cells were selected with medium containing 10 μg/mL of blasticidin (Invivogen; ant-bl-1) and then transduced with plenti X1/zeo shHrs, plenti X1/zeo shAlix, plenti X1/zeo shSyntenin, plenti X1/zeo shTsg101, plenti X1/zeo shCD63, or plenti X1/zeo shScramble. Doubly stable cells were selected with medium supplemented with blasticidin (10 μg/mL) and zeocin (200 μg/mL) for 2 weeks.

### Transfection

Plasmids containing wild-type human APP (APP^WT^)(pCAX APP 695) or human APP carrying the Swedish/Indiana mutations (APP^swe^)(pcAX APP Swe/Ind) were purchased from Addgene (Addgene plasmid #30137 and #30145 respectively) [66]. The Vps4a and Vps4a E228Q constructs were generously gifted from the laboratory of Dr. Nicholas Buchkovich (Pennsylvania State University) [44]. GFP-tagged APP^swe^ was created by inserting the pCAX APP Swe/Ind plasmid into the pENTR1A-GFP-N2 vector (Addgene #19364) between the Hind III and Ncol restriction sites using T4 ligase. Entry clones were then sequenced for verification, and recombined with the destination vector pQCXIZ CMV/TO DEST destination vector (Addgene #17401) using LR recombination (Invitrogen #11791–020) according to the manufacturer’s instructions, and as previously described [65].

HEK293 stably expressing silent shRNA constructs in 100 mm plates were induced by addition of 1 μg/mL of doxycycline 24 h before transfection with 5 μg of APP 695 or APP Swe/Ind plasmid using Lipofectamine 3000 transfection kit (Invitrogen, L3000015). Lipofectamine reagents were diluted in Opti-MEM medium (Gibco, 31,985–070) and added according to manufacturer’s instructions. Twenty-four hours after transfection, cells were harvested and lysed in RIPA buffer, as previously described [40]. Cell-conditioned medium was harvested for extracellular vesicle enrichment.

For experiments involving SH-SY5Y cells, a similar protocol was followed. However, SH-SY5Y cells were seeded in plates coated with poly-lysine according to the Poly-L-Lysine Cell Attachment Protocol (Sigma) to ensure adequate adherence. Twenty-four hours later, cells were induced with 1 μg/mL of doxycycline to promote shRNA expression. To differentiate the neuroblast-like cells into a phenotype resembling mature neuronal cells, serum-containing medium was aspirated 24 h after induction and replaced with serum-free medium. In addition, 10 μM of all-trans retinoic acid (Sigma, R2625) was added to cells, according to Shipley et al. [67] at the time of APP Swe/Ind plasmid transfection 24 h after induction. Forty-eight hours later, cell-conditioned medium was harvested for EV enrichment, and cell lysates were prepared as described below.

### Extracellular vesicle enrichment

Extracellular vesicles were isolated from cell-conditioned medium by modified differential centrifugation involving a polyethylene glycol (PEG) precipitation/concentration step as previously described and extensively characterized [40, 68–70]. Briefly, medium was collected and centrifuged serially (500 x g for 5 min; 2000 x g for 10 min; 10,000 x g for 30 min) to remove larger contaminants, or collect pellets for Fig. 1a. To enhance the concentration and yield of small EVs, supernatants following the 10,000 g spin were incubated with a 1:1 volume of 2× PEG solution (16%, wt/vol, polyethylene glycol, 1 M NaCl) overnight. The next day, solutions were centrifuged at 3214 x g for 1 h to obtain crude EVs. Pellets were resuspended in phosphate-buffered saline (PBS) before an ultracentrifugation wash at 100,000 x g for 70 min. EV pellets following ultracentrifugation were lysed in 2× nonreducing Laemmli sample buffer (4% SDS, 100 mM Tris-HCl [pH 6.8], 0.4 mg/mL bromophenol blue, 20% glycerol) for immunoblot analysis. Alternatively, pellets for nanoparticle tracking analysis were resuspended in particle-free PBS. For further purification and sub-population separation by floatation density gradient, EV pellets following the 100,000 x g spin were instead resuspended in 1.5 mL of 0.25 M sucrose buffer (10 mM Tris [pH 7.4]). Gradients (10–30%) were constructed as previously described in detail [40, 41, 43] using OptiPrep (Sigma, D1556). Following fractionation, densities of gradient separated fractions were estimated by measuring refractive indices of fractions with a refractometer (Refracto 30PX). Samples were then washed in PBS and pelleted again by ultracentrifugation at 100,000 xg for 2 h. Final pellets were resuspended in particle-free PBS for electron microscopy. A 1:1 solution of strong urea-containing lysis buffer (5% SDS, 10 mM EDTA, 120 mM Tris-HCl [pH 6.8], 8 M urea) with the addition of a protease inhibitor cocktail (Thermo, 78,438) was added to samples in fractions 2–6 for immunoblot analysis. EV characterization was performed in accordance with the minimal information for studies of EVs (MISEV) 2018 guidelines issued by the International Society for Extracellular Vesicles [71, 72].

### Electron microscopy

Electron microscopy was conducted on EV samples isolated in vesicle-rich fractions of the iodixanol density gradient purification step. Grids were prepared and images were obtained as previously described [73].

### Nanoparticle tracking analysis

Nanoparticle tracking was performed using a Malvern NanoSight LM10 instrument, and videos were processed using NTA 3.1 software as previously described [74].

### Immunoblot analysis

Whole-cell lysates were prepared by washing cells and then scraping them into cold PBS before pelleting at 1000×g for 10 min and lysis by radioimmunoprecipitation assay (RIPA) buffer as described previously [40]. To prepare all cell and EV lysates run under reducing conditions for SDS-PAGE, additional sample buffer (5×) also containing 0.2 M dithiothreitol (DTT) and 2% BME was added to samples. Equal protein of cell lysates measured by Pierce 660 nm Protein Assay (Invitrogen, 22,662) or equal volume of EV samples was loaded into an SDS 10, 12%, or 15% polyacrylamide gel. Western blot analysis was performed as described [40]. Ponceau S stain was used to visualize total protein. Blots were probed using the following antibodies: Alix (Q-19; Santa Cruz Biotechnology, 2171; Cell Signaling), Hsc70 (B-6; Santa Cruz), Tsg101 (C-2; Santa Cruz, 4A10; Genetex), CD63 (TS63; Abcam), Hrs (M-79; Santa Cruz), APP (LN27; Biolegend, 2452; Cell Signaling), Amyloid beta (8243, 2454; Cell Signaling), CD9 (P1/33/2; Santa Cruz, MM2/57; Millipore), Syntenin-1 (S-31; Santa Cruz), Flotillin-2 (H-90; Santa Cruz), GFP (600–101-215; Rockland), CD81 (H-121; Santa Cruz), Calnexin (H-70; Santa Cruz), CD71 (13,113; Cell Signaling), APP-CTF (A8717; Millipore), β-Amyloid 1–16 (6E10; Biolegend), rabbit anti-mouse IgG (Genetex, 26,728), rabbit anti-goat IgG (Genetex, 26,741), or goat anti-rabbit IgG (Fab fragment) (Genetex, 27,171). Blots were imaged using an Image Quant LAS4000 (General Electric) and processed with ImageQuant TL v8.1.0.0 software, Adobe Photoshop CS6, and CorelDraw Graphic Suite X5.

### Trypsin treatment of EVs

For experiments in Fig. 9c-d, PBS-suspended EVs from cells transfected with APP^swe^ were subjected to trypsin pre-treatment. Vesicles were incubated with 0.25% trypsin EDTA (Corning, 25–053-CI) or PBS with 2.21 mM EDTA (Thermo 1,861,275) for 20 min at 37 °C. EVs were then washed with PBS and ultracentrifuged at 100,000 x g to re-pellet the vesicles before lysis with 0.1% SDS and protein quantification. Treated vesicle proteins were analyzed by conventional western blot analysis or dot blot analysis.

### Immunofluorescence assay

HEK293 cells stably expressing shRNA plasmids targeting a scrambled control, Alix, or Syntenin-1 were seeded in 6-well plates on square glass coverslips. Cells were induced with 1 μg/mL doxycycline five hours later. Cells were transfected with 2 μg of APP Swe/Ind plasmid DNA using Lipofectamine 3000 (Invitrogen) 20–24 h after initial seeding. Twenty-four hours after transfection, medium was aspirated, and cells were gently washed with PBS. Cells were fixed on the coverslip in 4% paraformaldehyde for 10 min, then washed again in PBS before permeabilization in 0.2% Triton X-100 (in PBS) for 30 min at room temperature. A 0.2% Tween solution in PBS was prepared to make PBS-T. Cells were blocked in 5% goat serum/PBS-T for another 30 min at room temperature, then primary anti-APP antibody (2452; Cell Signaling) was added for a 3-h room temperature incubation. Cells were washed with PBS before secondary anti-rabbit antibody in 5% goat serum/PBS-T was added for 1 h at room temperature, then subsequently washed with PBS, and incubated with DAPI stain (Thermo, 62,248) in PBS for 10 min. Finally, cells were washed once more with PBS and mounted on a glass slide with mounting medium (4% propyl gallate, 90% glycerol in PBS) for confocal microscopy imaging. Confocal images were taken using a Zeiss LSM 880 microscope with 488-nm and 405-nm lasers and processed using Zen 2.1 Black software.

Primary neuronal cultures at days in vitro (DIV) 9 were stained with an antibody against microtubule associated protein 2 (Map2), a neuronal cell body and neurite marker. Cells were grown on poly-D-lysine-coated glass coverslips and were fixed in 4% paraformaldehyde and 4% sucrose in PBS for 20 min at room temperature, then blocked in a PBS solution containing 0.3% Triton X-100 (PBST) and 5% normal goat serum for 30 min at room temperature. Cells were then incubated in 1% normal goat serum in PBS containing anti-MAP2 antibody (17490–1-AP, ProteinTech, 1:250 dilution) at 4 °C overnight. The next day, sections were washed with PBS 3 × 5 min, followed by incubation with FITC-conjugated secondary antibody (4050–02, Southern Biotech, 1:1000 dilution) for 2 h at room temperature. After several washes with PBS, the coverslips were counterstained with 5 μg/mL DAPI (Sigma-Aldrich) in PBS for 5 min, washed in PBS for 5 min and mounted with Vectashield (Vector laboratories) to retard fluorescence fading. Images of cells were obtained using a Keyence BZ-X710 fluorescent microscope.

### Live cell imaging

For subcompartmental localization analysis of APP^swe^, shRNA-containing HEK293 cells were seeded into 35 mm glass bottom dishes (Greiner, 627,860) and induced with doxycycline the next day, as described above. Approximately 4 h later, cells were transfected with 2 μg of GFP-tagged APP using lipofectamine transfection reagent, and incubated for another 24 h. Staining with cellular compartment specific stains was performed as follows. Lissamine™ Rhodamine B 1,2-Dihexadecanoyl-sn-Glycero-3-Phosphoethanolamine, Triethylammonium Salt (rhodamine DHPE; Thermo Fisher; L1392) was used to stain endosomal compartments as previously described [75]. Briefly, stock DHPE was dissolved in chloroform for a concentration of 5 mg/mL. One μL of stock was resuspended in 50 μL of cold 95% ethanol, then further diluted 1:600 in cold PBS, before adding 200 μL to cells in a dropwise manner and incubating for 30 min. Cells were washed and double stained with Hoechst nuclear stain (5 μg/mL; 62,249; Thermo Scientific) before live-cell imaging. LysoTracker™ Red DND-99 (Thermo Fisher; L7528) was used to highlight lysosomal compartments by incubated cells with 75 nM of the LysoTracker stain before washing and adding Hoechst nuclear stain for 30 min. For Golgi staining, cells were incubated with CellLight® Golgi-RFP, BacMam 2.0 (15 particles per cell; Thermo Fisher; C10593) 16 h before washing, staining with Hoechst nuclear stain, and imaging. For ER staining, 1 μM final concentration of ER-Tracker™ Blue-White DPX (Thermo Fisher; E12353) was added to cells for 30 min before cells were washed and imaged. To visualize autophagic vacuoles within cells, Mono-dansylcadaverine (MDC; 30,432; Sigma) was added to cells at a final concentration of 50 nM for 30 min before washing cells and imaging as previously described [69]. All images were taken using a Zeiss LSM 880 microscope with 543-nm, 488-nm, and 405-nm lasers and processed using Zen 2.1 Black software.

### Neurotoxicity assay

Following growth in neurobasal A medium, primary cortical neurons were treated with 2 μg/mL puromycin, 0.25 μM purified oligomerized amyloid beta (see reactive oxygen species detection methods below), or 20 μg of EVs isolated from respective cell lines by PEG precipitation and subsequent ultracentrifugation wash. Forty-eight hours after treatment, medium was aspirated and cells were scraped into cold PBS and centrifuged at 300 xg for 2 min. Cells were resuspended in 100 μL of Annexin V binding buffer containing 0.25 μL Annexin V-FITC and 0.25 μL ViaStain™ propidium iodide (PI) stain (Biolegend, 422,201; Biolegend, 640,905; and Nexcelom, CSK-0112) then incubated in the dark for 10 min. Cells were centrifuged again at 300 g for 2 min, and resuspended in 500 μL of Annexin V binding buffer (without stains). Images were taken on a Keyence BZ-X710 fluorescent microscope.

Puncta Analyzer plugin was used as described previously [76] to quantify individual puncta for Annexin V and PI stained cells. Five 10x images per condition were analyzed with a minimum of 200 cells counted per group. With region of interest (ROI) selected as the full 10x image, the Puncta Analyzer plugin was used to analyze Red channel (PI) and Green Channel (Annexin V) following background subtraction (rolling ball radius of 50, without white background). Threshold remained consistent across groups, and the minimum puncta size was set to 4 pixels. Data are displayed as puncta per microscopic field normalized to the total number of neuronal cells per field.

### Undifferentiated hiPSC culture and cortical spheroid differentiation

Human iPSK3 cells were maintained in mTeSR serum-free medium (StemCell Technologies, Inc., Vancouver, Canada) on 6-well plates coated with growth factor reduced Geltrex (Life Technologies, Carlsbad, CA) as previously reported [77]. The cells were passaged by Accutase dissociation every seven days and seeded at 1 × 10^6^ cells per well of tissue culture treated 6-well plate (in 3 mL medium) in the presence of 10 μM Y27632 (Sigma) for the first 24 h [77–79]. For neural differentiation, human iPSK3 cells were seeded into Ultra-Low Attachment 24-well plates (Corning Incorporated, Corning, NY) at 3 × 10^5^ cells/well in 1 mL of differentiation medium composed of Dulbecco’s Modified Eagle Medium/Nutrient Mixture F-12 (DMEM/F-12) plus 2% B-27 serum-free supplement (Life Technologies). Y27632 (10 μM) was added during the seeding and removed after 24 h. On day 1, the cells formed spheroids and were treated with dual SMAD signaling inhibitors 10 μM SB431542 (Sigma) and 100 nM LDN193189 (Sigma) [77, 79, 80]. After 8 days, the cells were treated with fibroblast growth factor (FGF)-2 (10 ng/mL, Life Technologies) and cyclopamine (1 μM, Sigma) until day 24–30. Next day, the spheroids were re-plated to Geltrex-coated plates and grown for 3–5 days before the treatments.

### Reactive oxygen species detection

To prepare oligomers of the Aβ42 peptide, biotinylated Aβ42 (Bachem) was fully dissolved at 0.5 mg/mL in hexafluor-2-propanole (HFIP, Sigma) [77, 81]. HFIP Aβ (1-42) solution was dispensed at 10 μL into each siliconized Snap-Cap microtube. The microtubes were put in a desiccator to completely evaporate HFIP and thereafter stored at − 80 °C. Oligomer solutions were prepared freshly for each experiment. The stock was dissolved in 10 μL of DMSO (to 105 μM) and incubated for 3 h at room temperature. Oligomers of Aβ42 were added to the day 27–35 cortical spheroid cultures at 1 μM, as previous published [77, 81], and incubated for 48 h before analyzing. EVs (20 μg) harvested from control HEK293 cells, and cell lines expressing APP^WT^ or APP^swe^ in the absence or presence of concomitant shRNA targeting Alix or Syntenin-1 were added to spheroid cultures of the same maturity, then incubated for 48 h. Reactive oxygen species (ROS) detection was performed using Image-iT™ Live Green Reactive Oxygen Species Detection kit (Molecular probes), as previously described [82]. Briefly, the cells were harvested and washed in Hank’s Balanced Salt Solution, and incubated in a solution of 25 μM carboxy-H_2_DCFDA for 30 min at 37 °C. The samples were then washed and analyzed by flow cytometry. The cultures treated with Aβ42 oligomers were used as the positive control. The cultures that did not receive any treatment were used as the negative control.

### Data analysis and statistics

Statistical analysis was performed using the GraphPad Prism 8 (GraphPad Software, San Diego, CA) or Microsoft Excel with a significant threshold of *p* ≤ 0.05. All results are presented as means ± standard error of means (SEMs). The statistical significance of differences between means was assessed using the one-way ANOVA with a post hoc Tukey’s multiple comparison test or student’s t-test. Pearson correlation coefficient (PCC) was determined using an ImageJ colocalization plugin, with a minimum of 10 cells analyzed. Analysis was performed using GraphPad Prism 8 was used to determine significance using a one-way ANOVA with a post hoc Tukey’s multiple comparison test.

## Supplementary information

**Additional file 1.** Uncropped images of immunblots used in figures.

## Data Availability

The datasets used and/or analyzed during the current study are available from the corresponding author upon reasonable request.

## Abbreviations

APP: Amyloid Precursor Protein
CTFα: Alpha C-terminal fragment
CTFβ: Beta C-terminal fragment
Aβ: Amyloid beta
EVs: Extracellular vesicles
AD: Alzheimer’s disease
ESCRT: Endosomal sorting complexes required for transport
BACE: Beta-secretase
Hrs: Hepatocyte growth factor-regulated tyrosine kinase substrate
Stam: Signal transducing adaptor molecule
Tsg101: Tumor susceptibility gene 101
MVB: Multivesicular body
ILV: Intraluminal vesicles
WT: Wild-type
Swe: Swedish/Indiana mutation
KD: Knockdown
DMEM: Dulbecco modified Eagle medium
ACUC: Animal Care and Use Committee
RIPA: Radioimmunoprecipitation assay
PEG: Polyethylene glycol
PBS: Phosphate-buffered saline
MISEV: Minimal information for studies of EVs
TBS-T: Tris-buffered saline with Tween 20
DHPE: 1,2-Dihexadecanoyl-sn-Glycero-3-Phosphoethanolamine, Triethylammonium Salt
PI: Propidium iodide
MDC: Monodansylcadaverine
ROS: Reactive oxygen species
iPSK3: Human induced pluripotent stem cells
